# A Review of Potential Beneficial Effects of Honey on Bone Health

**DOI:** 10.1155/2019/8543618

**Published:** 2019-09-19

**Authors:** Mohd Amir Kamaruzzaman, Kok-Yong Chin, Elvy Suhana Mohd Ramli

**Affiliations:** ^1^Department of Anatomy, Faculty of Medicine, Universiti Kebangsaan Malaysia, Jalan Yaacob Latiff, Bandar Tun Razak, 56000 Cheras, Kuala Lumpur, Malaysia; ^2^Department of Pharmacology, Faculty of Medicine, Universiti Kebangsaan Malaysia, Jalan Yaacob Latiff, Bandar Tun Razak, 56000 Cheras, Kuala Lumpur, Malaysia

## Abstract

Bone remodelling is a complex and tightly regulated process. Disruption of bone remodelling skewing towards resorption will cause osteoporosis and increase the risk of fragility fracture. Honey is a natural product containing various bioactive ingredients with health benefits, especially polyphenols. Therefore, honey may be a novel dietary supplement to prevent osteoporosis. This review aims to summarize the current evidence on the effects of honey on bone health. The evidence reported so far indicates a skeletal-beneficial effect of honey in animal models of osteoporosis. However, the number of studies on humans is limited. Honey can protect the bone via its antioxidant and anti-inflammatory properties, primarily through its polyphenol content that acts upon several signalling pathways, leading to bone anabolic and antiresorptive effects. In conclusion, honey is a potential functional food for bone health, but the dose and the bioactive contents of honey need to be verified prior to its application in humans.

## 1. Introduction

Osteoporosis is characterized by low bone mass, deterioration of bone architecture, compromised bone strength, and an eventual increase in the risk of fracture. Based on the National Health and Nutrition Examination Survey III (NHANES III), more than 9.9 million Americans were diagnosed with osteoporosis and an additional 43.1 million experience low bone density [1]. Osteoporosis is a growing problem in Asia due to the rapidly increasing elderly population [2, 3]. Approximately 13% of the Mainland Chinese adults were estimated to suffer from osteoporosis, and the disease was more prevalent in women aged ≥50 years (40.1% versus 22.5% in men) [4]. The estimated number of osteoporosis patients in South Asian countries, like India, had increased from 26 million in 2003 to 36 million in 2013 [5]. Overall, by the year 2050, 50% of the global hip fractures among population of age ≥50 years will occur in Asia [5].

Oxidative stress has been hypothesized to be the contributing factors of many chronic diseases, including osteoporosis [6, 7]. Reactive oxygen species (ROS) directly promotes the formation of osteoclasts, the bone-resorbing cells, in a process mediated by receptor activation of nuclear factor-kappa-B (NF-*κ*B) ligand (RANKL)-RANK interaction [8, 9]. This signalling pathway involves redox-sensitive components, such as tumour necrosis factor receptor-associated factor 6 (TRAF6), Rac1 (a member of the Rho-GTPase subfamily), and nicotinamide adenine dinucleotide phosphate oxidases (NOX) [10]. Oxidative stress also mediates the induction of mitochondrial dysfunction apoptosis [11, 12] and decreases the differentiation and activities of osteoblasts, the bone-forming cells [13].

Age-related chronic inflammation also plays an essential role in the pathogenesis of osteoporosis by influencing bone remodelling [14]. In the presence of RANKL, the cytokines TNF-*α*, interleukins (IL)-6, and IL-1 will cause an excessive formation of osteoclasts while inhibiting osteoblast activities [15]. Proinflammatory cytokines like TNF-*α* also stimulates osteoclast development and increases the production of macrophage colony-stimulating factors (M-CSF) by bone marrow stromal cells (BMSCs) [16, 17]. They also suppress osteoblasts from releasing the RANKL decoy receptor, osteoprotegerin (OPG). This decoy primarily prevents the binding of RANKL with its corresponding receptor to stimulate osteoclastogenesis [15].

Calcium with or without vitamin D is the currently recommended prophylaxis for osteoporosis. For women of age ≥50 years, the recommended daily calcium intake is 1000 mg (dietary or supplementary) [18]. However, some studies suggested that excessive calcium supplementation is associated with cardiovascular events [19]. A meta-analysis revealed that calcium supplements without vitamin D significantly increases the risk of myocardial infarction (relative risk: 1.27, 95% confidence interval (CI): 1.01–1.59) [19]. The risk of cardiovascular events is predominantly observed in studies with the intakes of higher doses of calcium supplements (1000–2000 mg), whereas anything lower is deemed safe [21]. The Women's Health Initiative study showed that the relative risk for myocardial infarction in women taking calcium with or without vitamin D was 1.24 (95% CI: 1.07–1.45, *p* = 0.004) and 1.15 (95% CI: 1.03–1.27, *p* = 0.009), respectively, for the composite of myocardial infarction or stroke [19]. Barring the safety concerns of calcium supplementation, ensuring adequate dietary calcium intake is undoubtedly the first and the foremost step in osteoporosis prevention. However, there is a need to look for an alternative agent in preventing osteoporosis for those who are contraindicated to take calcium supplements.

Considering the involvement of oxidative stress and inflammation in the pathogenesis of osteoporosis, a functional food with properties that can counteract these processes may be a suitable prophylactic agent to prevent bone loss. Honey is one of the natural products with such properties. It is a sweet, viscous liquid substance produced mainly by a group of honeybees (from the genus *Apis*), which is best known worldwide due to the commercial production and human consumption for health maintenance [7]. It is stored in wax-form structures called honeycombs after being produced by honeybees from plants (floral nectar) through regurgitation, enzymatic activity, and water evaporation in the beehives. Apart from the nectar source, honeybees also collect secretions from insects (belonging to the genus *Rhynchota*) to produce honey called honeydew honey [22]. From nutritional view, it represents a unique source of natural macro- and micronutrients, including fructose and glucose, which make up the majority of the honey constituents. In addition to these, there is a wide range of beneficial minor constituents, especially phenolic compounds [23].

Honey also contains enzymes, amino acids, proteins, flavonoids, phenolic acids, organic acids, vitamins, and minerals in lower quantities [23]. Polyphenols, which covers a wide range of phytochemicals that are extensively studied by scientists for their health-promoting potential, can be found in almost all types of natural honey. Some of the polyphenols found in honey include flavonoids (such as apigenin, pinocembrin, kaempferol, quercetin, galangin, chrysin, and hesperetin) and phenolic acids (such as ellagic, caffeic, p-coumaric, ferulic, 3-hydroxybenzoic, chlorogenic, rosmarinic, vanillic, gallic, benzoic, and ascorbic acids) [24]. Most of these compounds work by interacting with each other to produce a range of synergistic antioxidant properties. Many published studies denote that honey possesses antioxidant, antibacterial, antiviral, anti-inflammatory, antiulcerous, immune-modulating, vasodilative, hypotensive, hypocholesterolemic, and antitumour activities [25].

In this review, evidence regarding the effects of honey on bone health derived from cellular, animal, and human studies was included. The information presented in this review will help to decide on the efficacy of honey as a functional food component in osteoporosis prevention. We will first summarize the evidence on the antioxidant and anti-inflammation properties of honey, before discussing its skeletal benefits.

## 2. Antioxidant Properties of Honey

The antioxidant activity in honey is primarily attributed to the presence of phenolic compounds and flavonoids [26]. Phenolic compounds exert antioxidant activities via several different mechanisms, such as free radical scavenging, hydrogen donation, singlet oxygen quenching, and metal ion chelation [27]. The total phenolic content (TPC) of honey can be determined through the Folin–Ciocalteu method. Physically, a higher TPC is indicated by the colour of the honey, whereby honey with a darker appearance tends to have a higher TPC as compared to the lighter one [28, 29]. Other measures to determine the antioxidant potential of honey include 1,1-diphenyl-2-picrylhydrazyl scavenging assay, 2,2′-azino-bis(3-ethylbenzothiazoline-6-sulphonic acid) assay, oxygen radical absorbance capacity assay, and ferric reducing antioxidant power assay [30].

The phenolic and flavonoid contents of honey can correct the redox imbalance in the body by counteracting the deterioration caused by oxidants such as oxygen, hydroxide, superoxide, and/or lipid peroxyl radicals [31]. The synergistic effect of exogenous nonenzymatic antioxidants in honey can be observed when they provide support for endogenous antioxidant enzymes, such as superoxide dismutase (SOD), catalase (CAT), and glutathione peroxidase (GPX), to eradicate ROS. One type of antioxidant may not be sufficient to ameliorate the damaging effect of oxidants. In contrast, it may be transformed into a pro-oxidant. In this regard, honey is advantageous by having several antioxidant constituents, which can assist each other to regenerate and prevent the formation of pro-oxidants [32, 33]. Additionally, both enzymatic and nonenzymatic antioxidant contents in honey may act at different cellular levels, by preventing oxidation of the macromolecules or stimulating gene expressions, which ultimately provoke an antioxidant response [31, 34]. Offsprings of rats fed with Tualang honey also had a higher GSH level and a lower lipid peroxidation level in their spinal cord tissue after being challenged with formalin-induced stress [35]. Apart from interacting with oxidants physically, polyphenols in honey can activate intracellular signalling cascades, such as phosphatidylinositol-4,5-bisphosphate 3-kinase (PI3K), protein kinase B (PKB)/Akt, tyrosine kinases, protein kinase C (PKC), and mitogen-activated protein kinases (MAPKs) [36].

## 3. Anti-Inflammatory Effects of Honey

Oxidative stress plays a pathogenic role in chronic inflammatory diseases [37]. The production of free radicals like hydrogen peroxide (H_2_O_2_) by leukocytes plays a role in inflammation, whereby it is responsible for the oxidative activation of NF-*κ*B. The NF-*κ*B then regulates the expression of various genes encoding proinflammatory mediators such as cytokines, chemokines, growth factors, and inducible enzymes [38]. Several proinflammatory mediators are released during an inflammatory response, including cyclooxygenase-2 (COX-2), IL-6, IL-12, interferon (INF-c), TNF-*α*, and inducible nitric oxide synthase (iNOS) [39]. The activity of iNOS is stimulated during inflammation by bacterial endotoxins such as lipopolysaccharide (LPS) and cytokines, including TNF-*α* and ILs, to enhance the production of gaseous free radical, nitric oxide (NO) [40]. Together, both H_2_O_2_ and NO may promote self-amplification of the inflammatory response, causing cellular damaging effects.

Evidence suggested that there are several possible anti-inflammatory mechanisms of honey. It inhibits the production of proinflammatory mediators, such as NO, prostaglandin E_2_ (PGE_2_), TNF-*α*, and IL-6 in studies of carrageenan-induced inflammation model in rats [40–42]. The inhibition of TNF-*α* and NO expression by honey *in vitro* was also observed [43]. In a dextran sodium sulfate-induced ulcerative colitis rat model, the honey-treated group had significantly improved macroscopic (stool) and microscopic scores (regular folding of colonic mucosa with epithelia, crypts, submucosa, musculosa, and serosa) as well as down-regulation of oxidative (SOD and GSH), inflammatory (TNF-*α* and iNOS), and apoptotic (caspase-3) markers [44]. Albumin denaturation inhibition, membrane stabilization, and proteinase inhibition assays indicated that flavonoid extracts from *M. beecheii* honey showed anti-inflammatory activity [45]. Honey also attenuated NF-*κ*B translocation to the nucleus, thus inhibiting I*κ*B*α* degradation with a subsequent decrease of inflammatory mediators COX-2 and TNF-*α* [46]. Each of the phenolic compounds in honey also possesses anti-inflammatory activity as shown in Table 1. For instance, garlic acid, a type of phenolic acids found in honey, exerts an anti-inflammatory action by suppressing COX-2, thereby inhibiting the production of proinflammatory cytokines and chemokines. The biological actions of NF-*κ*B, including the activation of transcription and DNA binding activity, are regulated by the acetylation of p65 [69]. Garlic acid inhibited p65 acetylation-dependent activation of NF-*κ*B and production of inflammatory markers. The low acetylation rate of p65 resulted in a complete loss of function of NF-*κ*B [70]. All of these bioactive compounds may act synergistically to contribute to the overall anti-inflammatory properties of honey.

## 4. Effects of Honey on Animal Models of Osteoporosis

### 4.1. Normal Animals

Honey is a commonly used sweetener consisting of carbohydrates, such as fructose, glucose, and raffinose. Despite not being as prominent as fructose and glucose in honey, various animal model studies have observed the calcium uptake-enhancing effects of raffinose [71, 72]. Calcium absorption in the rats following acute (2 days) and chronic feeding (8 weeks) of Pure U.S. Fancy White Honey had been studied. Ariefdjohan et al. found an increase in calcium absorption with an acute feeding of 500 mg (25.5%) or 800 mg (33.6%) of a honey, glucose-fructose mixture (17.1%) or high raffinose content (25.6%) as shown in calcium analysis from the right femur of the rats [73]. However, this calcium absorption-enhancing effect did not persist with a chronic feeding of 5% nor 10% honey in six-week-old growing rats [73].

Another study showed that the tibial calcium content was improved by the addition of honey at 20 ml/L to drinking water starting from day 28 until day 56 in heat-stressed broiler chickens [74]. This observation indicates that honey improves the calcium metabolism of the broilers [72]. There was also an improvement in the bone density of the broilers receiving honey supplementation, but the volume of their tibial bones was not affected by honey [74].

The long-term effects of feeding honeydew honey at the dose of 10% w/w diet for one year in comparison with sucrose and a sugar-free diet on weight gain, lipid profiles, and body composition (using dual-energy X-ray absorptiometry) in rats were examined [75]. The results showed that bone mineral density was significantly increased in the honey-fed rat group compared with the sugar-free diet group, but no differences in lipid profiles were found [75]. No differences in bone mineral density were observed between the honey- and sucrose-fed rats, although it was significantly higher in the honey-fed rats compared with the rats fed with a sugar-free diet. Honey also altered other metabolic parameters. The HbA1c levels were significantly reduced, and the HDL-cholesterol levels were significantly increased in the honey-fed group as compared with rats fed with sucrose or a sugar-free diet. Other indices in lipid profile among these three groups were similar. The favourable metabolic effects of honey may be due to the differences in the glycaemic index between honey and sucrose, as well as its antioxidant content. Thus, honey may be an osteoporosis-preventing agent for individuals who also suffer from poor glycaemic control or at risk for coronary heart disease.

### 4.2. Ovariectomized Rats

Postmenopausal osteoporosis occurs after the cessation of oestrogen production by the ovaries. The loss of the protective effects of oestrogen has a direct impact on the maintenance of bone health. It also gives rise to oxidative stress and inflammatory-mediated bone loss [76]. Ovariectomy is commonly used as a model of oestrogen deficiency due to the ability to mimic menopause in women [77]. Ovariectomized rats suffer from progressive bone loss similar to postmenopausal women [78]. Ovariectomized rats were used by Zaid et al. to determine the effects of Tualang honey on the female reproductive organs, tibial bone, and hormonal profile. Tualang honey is produced by Apis dorsett bees as they build beehives on the Tualang tree found in tropical rainforests. It was revealed that administration of Tualang honey at doses 0.2 g/kg and 1.0 g/kg for 2 weeks to ovariectomized rats significantly preserved the weight of the uterus and thickness of the vaginal epithelium, restored the morphology (trabecular thickness) of the tibial bones, and reduced the body weight compared to the untreated rats. The circulating levels of oestradiol and progesterone in the Tualang honey-treated groups were markedly lower than the untreated group. However, at the low dose (0.2 g/kg), Tualang honey increased serum-free testosterone levels in the ovariectomized rats, which might be protective against bone loss. It remained uncertain if Tualang honey promoted the conversion of oestrogen to androgen. The phenotypic changes induced by Tualang honey could be mediated by changes in hormone levels, especially androgens [79].

Another study by Zaid et al. compared the protective effects of Tualang honey and calcium supplementation on the trabecular bone structure in ovariectomized rats [80]. Rats receiving Tualang honey at 0.2 g/kg body weight for six weeks showed better bone structural parameters which was marked by an increase in bone volume (BV/TV), trabecular thickness (Tb.Th), and trabecular number (Tb.N) while decreasing intertrabecular space. The improvements caused by Tualang honey were superior to the 1% calcium supplementation in drinking water. It should be noted that these rats were not fed with calcium-deficient diet, so additional calcium supplementation did not seem to confer additional skeletal effects as compared to honey. Therefore, Tualang honey may be a viable prophylactic agent to prevent bone loss in postmenopausal women [80].

Manuka honey combined with *α*-cyclodextrin (Manuka Honey with Cyclopower™ (MHCP)) was developed to enhance the delivery of honey and improving its water solubility and stability [81, 82]. Katsumata et al. demonstrated that MHCP decreased the serum C-terminal telopeptide of type Ι collagen (a marker for bone resorption), femoral RANKL (stimulator of osteoclast formation), and nuclear factor of activated T-cells, cytoplasmic 1 mRNA expression (regulators of osteoclast formation) without any effect on uterine weight in ovariectomized mice. In addition, MHCP increased caecal bifidobacteria and bacteroid contents. These results suggested that the MHCP possesses prebiotic effects which potentiate the effects of honey by decreasing bone resorption in ovariectomized mice through suppressing inflammation [83].

Supplementation of Tualang honey at the dosage of 2 g/kg and 4 g/kg for 12 weeks in the study by Yudaniayanti et al. was found to increase biomechanical strength of the right femoral bone in ovariectomized rats [84]. This observation showed that honey could prevent bone fragility induced by oestrogen deficiency [84]. However, Tualang honey did not preserve lumbar calcium ash density in the study by Ibrahim et al. [85]. In fact, rats receiving 3 g/kg of honey possessed the lowest lumbar calcium ash density compared to the sham group [85]. This could have happened due to the dosage of honey that was rather high, leading to an induction of diabetes in the rats, which, in turn, promoted bone loss. However, this remained as a speculation as glucose homeostasis was not determined in their study.

### 4.3. Fracture Bone Healing

In a study investigating the fracture healing properties of honey and hydroxyapatite in rats, a significantly better fracture healing was seen through better bone formation, union, and remodelling on the radiograph score in the rats with honey autograft as compared to the other treatment groups at the second week. The rats treated with honey alone showed the poorest healing based on the radiograph score throughout the treatment period. Histopathological investigations revealed that the group treated with hydroxyapatite alone showed the poorest results in bone marrow formation compared to all other treatment groups. Therefore, honey and hydroxyapatite together appeared to exert better healing effects on bone defects as compared to using them separately [86].

Bone healing effects of honey was examined by Hajizadeh et al. using a mandibular bone defect healing model in rats [87]. A 2 × 2 mm defect was created with an extraoral incision at the mandibular angle. In the experimental group, the defect was filled with sterile honey, under the brand of Medihoney (Derma Sciences Inc., Princeton, USA). The defect was left unfilled in the control group. After two weeks, five samples of the experimental group were in the mineralization phase, while all samples of the control group were in the vascularization phase. After four weeks, the defects were filled in four samples of the experimental group, while all samples of the control group were in the mineralization stage. The histomorphometric assessment revealed that new bone formation in the experimental group was significantly better than the control group after two and four weeks. This study demonstrated that honey could increase and accelerate bone repair of small mandibular defects in rats [87].

Grayanotoxin (GTX) is a toxin found in the flower parts of plant species such as *Rhododendron* and Kalmia. It is present in rhododendron pollens and can also be found in honey produced from this regional plant. This toxin-containing honey is locally known as “mad honey”, and it is used for alternative therapies [88]. In a study comparing the effects of GTX-containing honey (mad honey) at 80 mg/kg/day, normal honey at 80 mg/kg/day, and propolis at 200 mg/kg/day on fracture healing, Sahin et al. showed that GTX-containing honey and propolis accelerated healing of artificial transverse fracture over the course of 30 days [89]. GTX and propolis may share similar therapeutic effects in healing bone fracture [89].

### 4.4. Combined Effects of Honey Supplementation and Exercise on Bone Health

Ooi et al. investigated the combined effects of eight-week jumping exercise and honey supplementation on bone health in rats [90]. The honey (type and composition not indicated) was orally supplemented to the rats at the dosage of 1 g/kg body weight/day via oral gavage, 30 min before the jumping exercise. Each rat was subjected to the exercise for a five-minute duration per day for five days per week. The combination regime elicited superior effects on the tibial bone geometry and mechanical properties as compared to the jumping exercise or honey supplementation alone in rats. However, there were no statistically significant differences in bone mineral density among the experimental groups [90], which might require more time to observe an effect.

Tavafzadeh et al., through three separate studies, investigated the effects of jumping exercises in combination with honey supplementation on bone properties in young female rats [91–93]. Each rat was subjected to the exercise for five minutes per day for five days per week. Tualang honey was orally supplemented to the rats at the dosage of 1 g/kg body mass/rat/day via force-feeding, 30 min prior to the jumping exercise for a duration of eight weeks [91, 92], while another study by the Tavafzadeh et al. [93] lasted for 8 and 16 weeks. From the first study, they found that the combination of honey and jumping exercise elicited more prominent beneficial effects on the tibial and femoral bone. This was shown by the increase in tibial wet and fat-free dry weights, tibial and femoral maximal load, tibial midshaft minimum diameter, and femoral midshaft maximum diameter compared to either jumping exercise or honey supplementation alone in young female rats [91]. In the second study with the same method, the combined regime significantly reduced bone resorption and enhanced antioxidant status indicated by lower serum C-terminal telopeptide of type 1 procollagen and higher serum total antioxidant status, respectively, but did not alter oxidative stress marker [92]. In the third experiment, Tavafzadeh et al. showed that continuous 16 weeks of combined jumping exercise and honey supplementation caused more beneficial effects on the tibial bone. This was evidenced by greater tibial maximum force, energy, proximal total bone density, proximal trabecular bone density, midshaft cortical bone density, cortical area, and midshaft cortical moment of inertia in the rats receiving combinational therapy as compared with individual therapy [93]. Moreover, the beneficial effects of the combinational therapy on the tibial bone properties could still be maintained even after eight weeks of cessation of exercise and supplementation [93].

Mosavat et al. investigated the effects of high and low-intensity jumping exercise combined with honey on bone and gonadotrophins [94]. The low-intensity jumping exercise was defined as 20 jumps per day for five days/week for eight weeks, while the high-intensity jumping exercise was defined as 80 jumps per day for five days/week for eight weeks. The results showed that honey supplementation combined with high-intensity exercise invokes a greater increase in the tibial and femoral mass, serum total calcium, and alkaline phosphatase (ALP) levels. The hormonal changes were not observed in the combined honey and exercise groups. Serum luteinizing hormone concentrations were significantly greater in honey combined with the low- and high-intensity exercise group compared with the high-intensity exercise-alone group [94]. Mosavat et al. further evaluated the stress hormones and showed that the serum cortisol level was higher, while the serum progesterone level was lower in the group undergoing low- and high-intensity exercise alone compared with the control group [94]. In contrast, the combined honey plus exercise group had significantly lower serum cortisol and higher serum progesterone levels compared with the exercise alone group, which signifies the role of honey in regulating hormonal balance required for normal bone remodelling. For the reproductive system in these rats, the relative weight of the excised uterus was significantly greater in the honey plus low-intensity exercise group compared to the control group, indicating that it may have oestrogenic properties [95].

## 5. Human Study

### 5.1. Effects of Honey versus Hormonal Replacement Therapy

In a randomized controlled trial, 79 healthy postmenopausal women aged 45–60 years who were naturally menopausal for more than one year, not on hormonal replacement therapy (HRT) for more than three months and with a body mass index 18–35 kg/m^2^, were selected to test the effects of Tualang honey (dose of 20 g/day) and HRT (contain 1 mg oestradiol valerate and 5 mg dydrogesterone) on cardiovascular risk factors, hormonal profiles, and bone mineral density. It was found that there were no significant differences in the bone density, blood sugar level, and lipid profile between the two treatment groups at the end of the four-month study period [96]. This study was limited by its small sample size without involving all various ethnicities, a single dose of honey, lack of data on the effect of honey on postmenopausal symptoms, and short duration of the study.

Oxidative stress induced by oestrogen deficiency is postulated as one of the mechanisms leading to postmenopausal osteoporosis. Shafin et al. compared the effects of oestrogen-progestin therapy and Tualang honey in postmenopausal women [97]. In their study, Tualang honey supplementation for 16 weeks in postmenopausal women resulted in reduced oxidative stress as indicated by an increase in GPX or CAT activities and a decrease in 4-hydroxynonenal (4-HNE) level, respectively, at an extent comparable to those who received oestrogen-progestin therapy [97]. This oestrogenic property of honey could protect against postmenopausal osteoporosis.

### 5.2. Effects of Combined Honey Supplementation and Exercise

A study was conducted by Ooi et al. to investigate the effects of combined aerobic dance exercise and honey supplementation on bone turnover markers [99]. A honey drink, prepared from 20 g Gelam honey diluted in 300 mL plain water, was consumed by the subjects in the honey group and honey plus aerobic dance exercise group, for seven days/week for a total of six weeks. The subjects in the honey plus aerobic dance exercise group and aerobic dance alone group were required to undergo aerobic dance sessions for three sessions per week, one hour per session. The results suggested that combined intervention caused a greater increase in alkaline phosphatase compared to aerobic dance exercise or honey supplementation alone [99]. In another study, the researchers investigated the effects of aerobic dance exercise plus Gelam honey supplementation on metabolism markers and muscular performance in adult women. It was found that the combination of aerobic dance exercise and honey supplementation suppressed the serum C-terminal telopeptide of type 1 collagen resulting from exercise, possibly enhancing the lower limb muscular power in sedentary women [99]. This finding was consistent with the previous animal study, which showed that the combined regime significantly reduced bone resorption [92].

## 6. Conclusion

**Figure 1:**
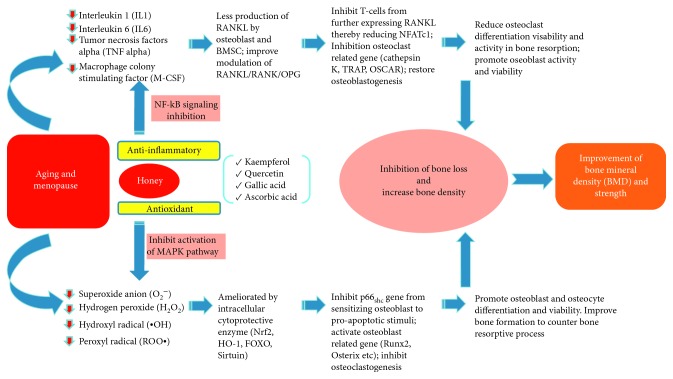
Potential effects of honey on bone health. Menopause and ageing give rise to oxidative stress and chronic low-grade inflammation, which cause bone loss. Phytochemical components found in honey, such as quercetin, kaempferol, gallic acid, and ascorbic acid, exert antioxidant and anti-inflammatory action by inhibiting activation of MAPK pathway and NF-*κ*B signalling, respectively. This action will prevent the formation of osteoclasts and favour bone formation by osteoblasts, subsequently preserving bone density.

The literature shows that honey has promising skeletal-beneficial effects in preventing osteoporosis. Many types of honey have been shown to prevent bone loss in various animal models via its high antioxidant and anti-inflammatory properties (Figure 1). Nevertheless, the efficacy of honey seems to be dose-dependent, whereby very high or low dosage will not produce desirable results on the skeleton. We cannot conclude a single effective dose due to the heterogenicity of the study design and honey types. It should be acknowledged that the composition of honey varies according to types and commercial brands and not all will have similar effects to the bone. Types, purity, and chemical constituents need to be considered before applying honey for any health purposes. Nevertheless, this review can serve as guidance for choosing the appropriate honey and its doses in osteoporosis prevention. The limited number of human studies suggested that effort needs to be made to validate the effects of honey in human, especially postmenopausal women. Particularly, long-term data are needed to show that honey consumption can lead to decreased bone loss and fracture risk.

## Figures and Tables

**Table 1:** Anti-inflammatory properties of common phenolic compounds found in honey.

Phenolic compound	Anti-inflammatory property	Reference
Ellagic acid	↓ PGE_2_ synthesis by inhibiting COX-2 and NF-*κ*B pathway ↓ expression of inflammatory mediators (TNF and NO)	[40] [43]
Gallic acid	↓ LPS-induced NO, PGE_2,_ and IL-6 production in RAW 264.7 cells ↓ MAPK/NF-*κ*B pathway by activating Akt/AMPK/Nuclear factor erythroid 2-related factor 2 pathway in RAW264.7 cells ↓ expression mRNA expression of NF-*κ*B and IL-6.	[47] [48] [49]
Caffeic acid	↓ LPS-induced NF-*κ*B activation ↓ iNOS and COX-2 production but ↑ haem oxygenase-1	[50] [51]
Ferulic acid	↓ production of TNF-*α* and IL-6 in RAW264.7 cells ↓ MAPK signalling pathways in rat with acute respiratory distress syndrome ↓ mRNA expression of IL-6, IL-1*β,* and TNF-*α* but ↑ IL-10 in male prenatally-stressed rat offspring	[52] [53] [54]
Syringic acid	↓ the inflammatory cells (eosinophil, neutrophil, macrophage, and lymphocyte) and production of inflammatory markers (IL-4, IL-5, IL-13, and TNF-*α*) in mice	[55]
Ascorbic acid	↓ the levels of hs-CRP, IL-6, fasting blood glucose, and triglyceride in hypertensive and diabetic obese adults	[56]
3-hydroxybenzoic acid	↓ the production of IL-1*β*, IL-6, and TNF-*α* by suppressing phosphorylation of MAPKs and NF-*κ*B p65 nuclear translocation in LPS-induced microglia.	[57]
Vanillic acid	↓ the mRNA expression of TNF-*α* and IL-1*β*, as well as protein production of iNOS/COX-2 ↓ LPS-induced degradation of I*κ*B*α* and nuclear translocation of NF*κ*B	[58]
Chlorogenic acid	↓ the expression of TNF-*α* and IL-1*β* but ↑ the expression of anti-inflammatory cytokine IL-10 in mouse intoxicated with neurotoxin	[59]
Coumaric acid	↓ the production of IL-8 in cigarette smoke extract-stimulated A549 cells	[60]
Rosmarinic acid	↓ the expression of inflammatory markers (COX-2, PGE_2_, IL-1*β*, MMP-2, and NO) in rats with neuropathic pain	[61]
Genistein	↓ colonic production of IL-1*β* and IFN-*γ* associated with the TLR4/NF-*κ*B signal	[62]
Luteolin	↓ LPS-triggered secretion and relocation of high mobility group B-1 and LPS-induced production of TNF-*α* and NO in macrophages	[63]
Naringenin	↓ RANKL-induced activation of NF-*κ*B by suppressing RANKL-mediated IkB-*α* degradation in RAW264.7 cells	[64]
Apigenin	↓ signal transducer and activator of transcription (STAT) 3-NF-*κ*B signalling of mice with inflammatory bowel disease and colorectal-associated cancer	[65]
Myricitrin	↓ osteoclastogenesis through the suppression of the NF-*κ*B signalling pathway and mitogen-activated MAPK pathways in RAW264.7 cells	[66]
Kaempferol	↓ the expression of proinflammatory cytokines (TNF-*α*, IL-1*β*, and IL-8) and the production of IL-8 in AGS cells	[67]
Quercetin	↓ the production of NO, IL-6, MCP-1, IP-10, RANTES, GM-CSF, G-CSF, TNF-*α*, leucocytes inhibitory factors, LIX, and vascular endothelial growth factors as well as calcium-STAT pathway in dsRNA-induced RAW 264.7 cells	[68]

Note: ↑, increase; ↓, reduce.
